# Native Medicines Used by the Pioneers

**Published:** 1916-10

**Authors:** 


**Native Medicines Used by the Pioneers.** William M. Johnson, of Shawnee Mission, Kan., Med. Herald.

In the earlier days of this country his father and mother were missionaries. The nearest doctor was in Independence, seventeen miles away. So in cases of sickness they depended on home remedies more than anything else.

The inside lining of chicken gizzard was much used for indigestion and intestinal disorder. At that time they used this by making it into a tea.

This was over 35 years ago, and this remedy, dried and powdered, has since become well known as ingluvin, but in this form is not so effective as the fresh. Boneset tea was used in fevers, ague and indisposition. It was the greatest thing to make you sweat. All families gathered large quantities of it in the early fall and put it away for winter use. He has seen men perspire thru the mattress under its effects.

White Root.—This was what his father depended on more than any one thing in treating the Indian children for colds, making a tea of it.

White oak bark was used when they wanted a strong astringent.

Black walnut bark is a purgative and a very powerful one. The sap that runs from a scar on the bark dries and forms a soft mass. A small pill made from this is a strong purge.

Hoarhound grew wild in large quantities. When in bloom the bees would make honey of it; this they would save separate and use in colds and sometimes make into candy.

Sumach.—The Indians used to eat the new growth of this for stomachache and fever. It is very healing to a sore throat and greatly relieves catarrh. He still makes almost every fall a cough syrup of sumach berries, walnut bark, wildcherry bark and hoarhound.

Blackberry in a cordial was counted a sovereign remedy for summer complaint in children, and if the berries were not to be had a tea from the root was used.

Nightshade with cream made a fine poultice for poison oak. The dried leaf of the jimson weed, or stramonium, was smoked for coughs and asthma, and was much used as a strong relaxing poultice.

Dog fennel was a common counter-irritant used as a poultice and will draw a strong blister.

May apple (mandrake) root as a purgative and tonic was the basis of all early ague and chill medicine. The thought of the taste of a threatened tea made from this often cured laziness blamed on the ague.

Elder bark extracted in suet had great virtue as a salve.

Dried elder flowers were popular for fevers and as a tonic. Common plantain leaf had much use as a poultice.

The infusion of old tobacco pipe as emetic and purgative was the dependence of every old-time doctor and granny.

The process of ''smoking them out'' with chicken feathers burning on hot coals under the bed was the final cure, all used with great ceremony, when diagnosis was uncertain and all other remedies had failed.

